# Eukaryotic Life Inhabits Rhodolith-forming Coralline Algae (Hapalidiales, Rhodophyta), Remarkable Marine Benthic Microhabitats

**DOI:** 10.1038/srep45850

**Published:** 2017-04-03

**Authors:** Sherry Krayesky-Self, William E. Schmidt, Delena Phung, Caroline Henry, Thomas Sauvage, Olga Camacho, Bruce E. Felgenhauer, Suzanne Fredericq

**Affiliations:** 1 University of Louisiana at Lafayette, Lafayette, Louisiana, 70504-3602, USA; 2 Smithsonian Marine Station at Fort Pierce, Fort Pierce, Florida, 34949, USA

## Abstract

Rhodoliths are benthic calcium carbonate nodules accreted by crustose coralline red algae which recently have been identified as useful indicators of biomineral changes resulting from global climate change and ocean acidification. This study highlights the discovery that the interior of rhodoliths are marine biodiversity hotspots that function as seedbanks and temporary reservoirs of previously unknown stages in the life history of ecologically important dinoflagellate and haptophyte microalgae. Whereas the studied rhodoliths originated from offshore deep bank pinnacles in the northwestern Gulf of Mexico, the present study opens the door to assess the universality of endolithic stages among bloom-forming microalgae spanning different phyla, some of public health concerns (*Prorocentrum*) in marine ecosystems worldwide.

Coralline red algae are calcifying multicellular seaweeds that are ubiquitous in marine ecosystems worldwide. Because they precipitate calcium carbonate within their organic cell walls as Ca(Mg)CO_3_, a highly soluble, high-magnesium calcite polymorph^1^,^2^,^3^, coralline red algae are currently popular experimental subjects of many ecological and mineralogical studies that address the potential effects of ocean acidification and global warming on calcifying organisms^2^,^4^,^5^,^6^. It is well known that the CaCO_3_ in the cell walls provides the coralline algae several advantages such as rigidity, skeletal strength, protection from grazing and boring animals, and enhanced survivorship by increasing resistance to wave action^7^. Less well known, however, is the nature of roundish cellular inclusions present within individual calcium carbonate-lined coralline cells. There is no consensus about the nature of such inclusions that have either been referred to as floridean starch^8^, chloroplasts^9^ or bacteria^10^,^11^,^12^. We observed similar omnipresent concentric inclusions in cells of coralline algae forming rhodoliths (literally “red stones” or maërl) with the scanning electron microscope (SEM). The hypothesis that these inclusions were floridean starch was quickly rejected when iodine starch reaction turned negative. Likewise, their large size excluded the concentric structures from being chloroplasts or bacteria^13^. The main objective of this study was to resolve the true nature of the cellular inclusions in rhodolith-forming corallines using a suite of microscopy, culturing, and DNA sequencing tools.

Rhodoliths are free-living, benthic, marine calcium carbonate nodules of various sizes and origins that are predominantly accreted by crustose coralline red algae (CCA)^14^,^15^ (Fig. 1A). Water motion (waves, currents) is critical in the maintenance of rhodoliths to grow unattached and unburied by sediments^16^ with their periodic rotation (rolling) allowing light exposure on all sides and limiting fouling^17^,^18^. As an essential component of photosynthetic communities, rhodoliths produce oxygen, consolidate substrata, and function as autogenic ecosystem engineers^19^. The deposition of CaCO_3_ by marine algae is an essential process in the global carbon cycle^20^ and rhodoliths are recognized as foremost carbonate builders^21^. Furthermore, corallines are one of the major producers of dimethylsulfoniopropionate (DMSP)^22^, which, upon being metabolized by algal-associated bacteria, produces volatile compounds such as dimethyl sulfide (DMS). The latter has direct effects on the global sulfur cycle and global climate change^23^. Rhodoliths also exude organic matter^24^ used by co-inhabiting prokaryotes, which in turn cycle key biogeochemical elements necessary for these primary producers and other eukaryotic rhodolith colonizers.

Prior to the 2010 Deepwater Horizon oil spill (DWH), extensive rhodolith beds covering the flanks and tops of hard bank pinnacles offshore Louisiana in the NW Gulf of Mexico, such as Ewing Bank (vicinity of 28°05.7′N, 91°01.2′W), typically sustained a lush diversity of encrusting, filamentous and fleshy macroalgae growing on the rhodoliths’ surface^25^. In contrast, after DWH the macroalgae covering these rhodoliths disappeared and most rhodoliths themselves appeared bleached, and fully or partially denuded of surface macroalgae, a situation that has persisted in the field as of September 2014, our last expedition to Ewing Bank. Bare rhodoliths, along with *in situ* seawater, were brought back to the laboratory and placed into 75-liter microcosms. After three weeks in the microcosms, diverse macroalgal growth emerged from the rhodoliths’ surface, a reflection of the algal community present at Ewing Bank prior to DWH^25^,^26^. Many taxa subsequently reached sexual maturity and life cycle completion. It was newly hypothesized that since macroalgae emerged from the surface of the bare rhodoliths, the latter may serve as seedbanks^25^,^26^, a hypothesis subsequently confirmed by metabarcoding^27^.

## Results and Discussion

**Figure 1:**
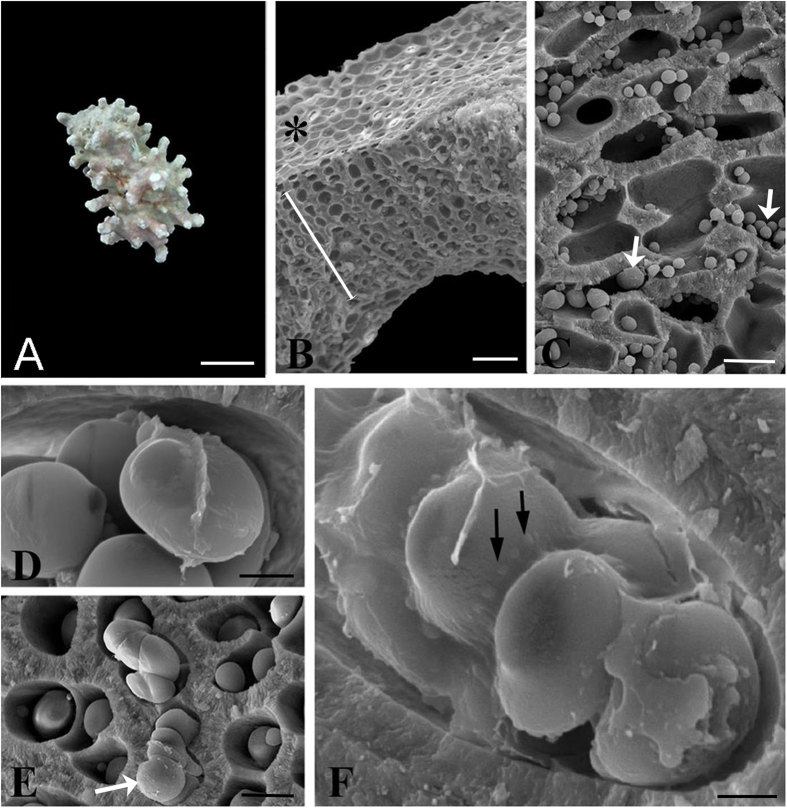
Newly documented endolithic life history stages of *Prorocentrum lima* dinoflagellates within calcified cell lumens of *Lithothamnion* sp. 1 rhodoliths from Ewing Bank, Sackett Bank and the Florida Middle Grounds, Gulf of Mexico. (**A**) [sample 5] Rhodolith habit, scale bar = 6.5 mm. (**B**) [sample 2] SEM micrograph of a cross section through *Lithothamnion* sp. 1, * = epithallus (1 cell layer); white line = perithallus (~12 cell layers) showing cellular inclusions (white arrows) within the perithallial cells, scale bar = 36 μm. (**C**–**E**) [samples C = 3, D & E = 9] SEM micrographs of *Lithothamnion* sp. 1 perithallial cells containing endolithic inclusions that display dinoflagellate characteristics, scale bars, C = 9 μm, D = 3 μm, E = 2.5 μm (**F**) Dinoflagellate stages showing blebs, *i.e*. organized membrane projections (black arrows), scale bar = 1 μm.

Scanning electron microscopy (SEM) images revealed aggregations of *Prorocentrum*, a well-characterized bloom-forming genus of benthic dinoflagellates^28^,^29^,^30^,^31^,^32^, at the surface of *Lithothamnion* sp. 1 rhodoliths in the microcosms. Field emission scanning electron microscopy (FESEM) images also revealed in fractured cross sections that the inner cells of *Lithothamnion* sp. 1 rhodoliths harbored abundant spherical, oval and disk-shaped structures (Fig. 1B–E, arrows) occupying the lumen of the coralline’s calcified perithallial cells. The possibility that the concentric structures were endolithic cellular stages that were part of a dinoflagellate life cycle was thus further explored. Subsequently, FESEM was used which indicated that some of the concentric structures were clustered together and covered by a membrane (Fig. 1D–F) displaying projecting blebs (Fig. 1F, arrows). Even though corallines belong in the Supergroup Plants^33^, the calcium carbonate-encrusted cell lumens are hard and “stony,” the endophytic (“inside plant”) nature of the inclusions can be interpreted as endolithic (“inside stone”).

**Figure 2:**
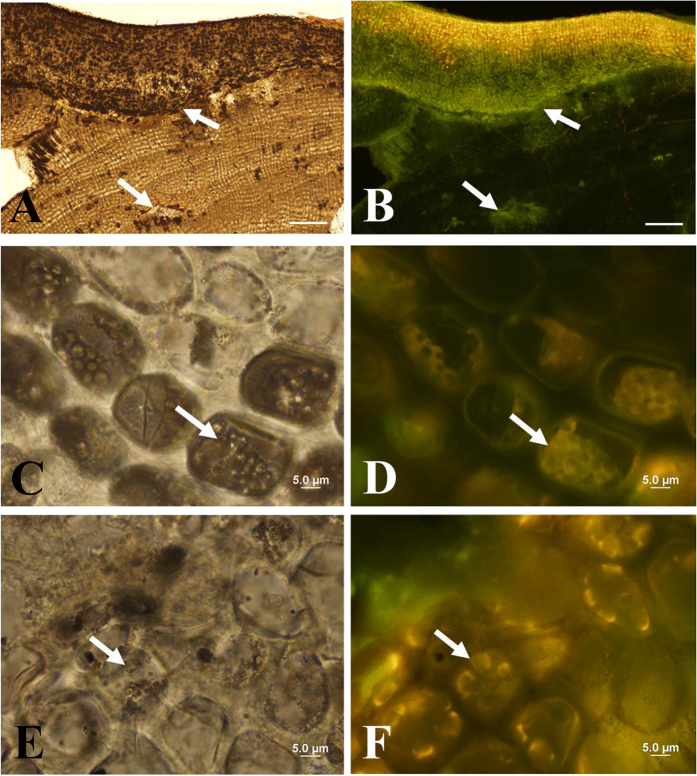
Light (LM) and Fluorescence micrographs of cross section of *Lithothamnion* sp. 1 rhodolith [sample 26] and endolithic dinoflagellate life history stages. (**A**,**C**,**E**) Light micrographs and (**B**,**D**,**F**) corresponding fluorescence micrographs. Dinoflagellate cells (white arrows) fluoresce green and *Lithothamnion* cells fluoresce yellow. Semi-thin sections (24 × 46 mm polished to ≈30 microns) through the rhodoliths revealed dark brown cells in the upper part of the sections with light microscopy (LM) (i.e., perithallial and upper epithallial cell layers of the corallines) (**A**), with the same cells in a fluorescence image seen as yellow (**B**). In contrast, in the deeper cell layers of the coralline (i.e., the lower perithallial cells, **C**,**E**), green light is emitted (**B**). Corresponding (**B**) shows the bright yellow/orange autofluorescence of the photosynthetically active coralline red algae, and a clear green region where the cellular inclusions are located. (**D**,**F**) are magnified views of the same sample displayed in (**B**) showing autofluorescence of the cellular inclusions enclosed within calcium carbonate-lined coralline cells. (**F**) points to 1 structure inside of a cellular inclusion of which a group is shown in (**D**). The arrow in (**B**) points to the area in the coralline where the cellular inclusions were found. Scale: **A** & **B** = 100 μm, **C**–**F** = 5 μm.

Epifluorescence microscopy was employed owing to the fact that when excited by blue light, dinoflagellates emit green autofluorescence that has been used to visualize and enumerate these organisms^34^ in the phytoplankton or to track their cysts in ocean sediments^35^ as well as their presence in the phytoplankton^36^. Red algae also autofluoresce, but the emitted wavelength when excited by blue light is red or yellow^35^. Semi-thin sections (24 × 46 mm polished to ≈30 microns) through the rhodoliths revealed dark brown cells in the upper part of the sections with light microscopy (LM) (i.e., the perithallial and upper epithallial cell layers of the corallines) (Fig. 2A), with the same cells in a fluorescence image seen as yellow (Fig. 2B). In contrast, in the deeper cell layers of the coralline (i.e., the lower perithallial cells, Fig. 2C,E, magnified views of Fig. 2A), green light was emitted (Fig. 2B). Corresponding Fig. 2B shows the bright yellow/orange autofluorescence of the photosynthetically active coralline red algae, and a clear green region where the cellular inclusions are located. Figure 2D and F are magnified views of the same sample displayed in Fig. 2B showing autofluorescence of the cellular inclusions enclosed within calcium carbonate-lined coralline cells. However, because of DNA evidence (see below), these green fluorescing cells are likely to be dinoflagellate in nature. These cellular inclusions pointed to in Fig. 2D are not coralline in nature, and the green fluorescing cells are not floridean starch, not bacteria, not individual chloroplasts outside of the cellular inclusions, or are not degraded coralline chloroplasts (because these structures are found within a membrane-enclosed structure). Note that the arrow in Fig. 2F points to one structure inside a cellular inclusion of which a group is shown in Fig. 2D. The arrow in Fig. 2B points to the area in the coralline where the cellular inclusions were found intracellularly. The yellow nature of this autofluorescence, rather than typical green fluorescence of dinoflagellates, may be the result of two phenomena: 1) the autofluorescence of pigments other than chlorophyll *a* or *b*, and 2) the autofluorescence from pigments that have been exposed to various fixation chemicals and processes known to affect and sometimes compound the fluorescent signal.

**Figure 3:**
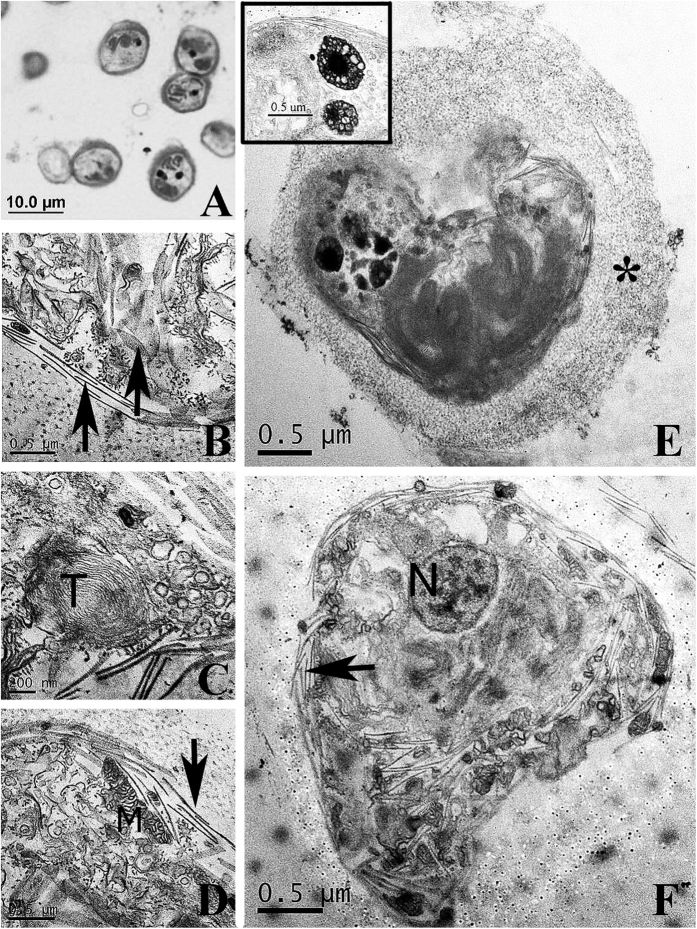
Endolithic dinoflagellate life history stages pulled from rhodolith [sample 3a and 5] (**A**) Light and (**B**–**F**) Transmission electron (TEM) micrographs. (**A**) Isolated endolithic cellular inclusions stained with Osmium Tetroxide (OsO_4_) and Methyl Blue. (**B**) Putative thecal plate primordia (black arrows in **B**,**D** & **F**). (**C**) Concentric thylakoid membranes (T). (**D**) Mitochondria (M). (**E**). Layer of extracellular matrix surrounding cell (*) and collecting pusules (insert E). Arrow points to pusule location intracellularly. (**F**). Condensed chromosomes in the dinokaryon (**N**).

Rhodoliths were not decalcified for SEM microscopy, but slightly for use with transmission electron microscopy (TEM). After TEM fixation, a piece of the *Lithothamnion* sp. 1 rhodolith that had been used for FESEM was partially decalcified by decreasing the time of the reaction (Fig. S1A). Partial decalcification was critical for capturing the clusters of cells from within the coralline medullary (i. e. perithallial) cells (Fig. S1B). The *Lithothamnion* upper epithallial cell layers were removed and the precipitate from inside the rhodolith was contrasted further in Osmium Tetroxide (OsO_4_), dehydrated, embedded in resin, sectioned and viewed using LM (Fig. 3A) or (TEM) (Fig. 3B–F). This revealed small clusters of cells, similar to the clusters observed in FESEM, throughout the resin block (Fig. 3A). Structures we interpret as putative thecal plate primordia (Fig. 3B), thylakoid membranes (Fig. 3C), mitochondria (Fig. 3D), collecting pusules (Fig. 3E insert), and condensed chromosomes in the dinokaryon (Fig. 3F) are typical of dinoflagellates^37^,^38^. A thick layer of extra-cellular matrix was also observed surrounding each cell (Fig. 3E). Thecal plate primordia (black arrows) are also displayed in Fig. 3C–F.

To confirm whether the putative dinoflagellate stages growing endolithically within the *Lithothamnion* sp. 1 rhodolith cells could be linked specifically with any of the free-living dinoflagellates (i.e. *Prorocentrum, Gambierdiscus, Coolia, Amphidinium*) present in the water column of the Ewing Bank microcosms, total DNA was extracted separately from material removed from both the interior of the *Lithothamnion* perithallial cells and from single, free-living cells present in the microcosms. These extractions were successfully PCR-amplified using dinoflagellate-specific *cob1* primers^39^. Generated *cob1* sequences originating from both inside the rhodolith and from free-living *Prorocentrum* cells were identical to each other, and when BLASTed on GenBank were an exact match to *Prorocentrum lima* (Fig. S2), a common coastal dinoflagellate of public health concern^29^,^30^. A *Prorocentrum* phylogenetic *cob1* tree constructed by RAxML (Fig. S2) revealed rhodolith-associated *Prorocentrum* cells nested within a *Prorocentrum* clade that corresponds to *P. lima* strain 1966 (https://ncma.bigelow.org) with strong bootstrap support (BS = 98).

**Figure 4:**
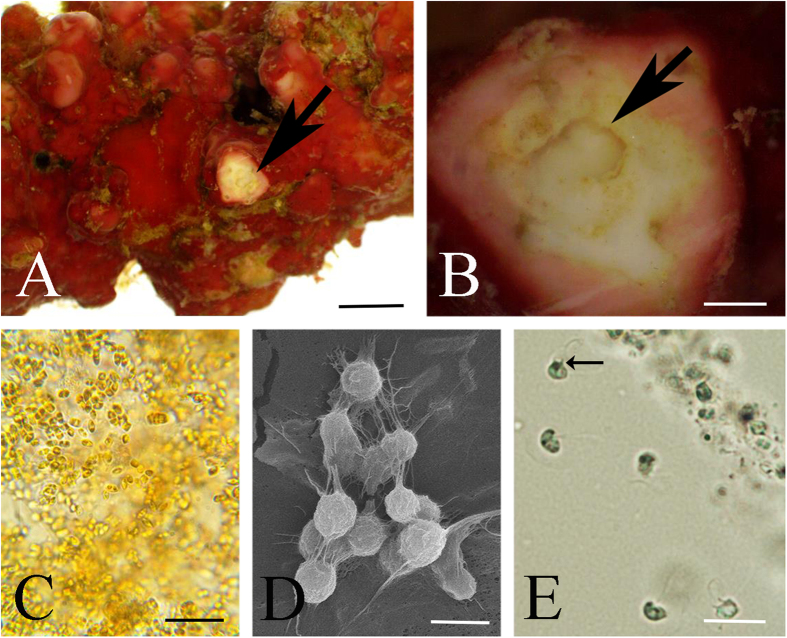
(**A**) *Lithothamnion* sp. 2 rhodolith [sample 3b] with newly documented endolithic stages of *Ochrosphaera verrucosa* haptophytes, scale bar = 1 mm. (**B**) Shaved area of *Lithothamnion sp. 2* rhodolith from Ewing Bank exposing brown patches of non-flagellated *O. verrucosa*, scale bar = 3 mm. (**C**,**D**) Cultures of non-flagellated cells isolated from within the rhodolith shown with light microscopy (**C**) and SEM (**D**), scale bars C = 700 μm, D = 2.5 μm. (**E**) Cultures shown in C,D subsequently developed into biflagellated cells with a rudimentary haptoneme (arrow), scale bar = 4.5 μm.

Just as clusters of dinoflagellate stages were associated within rhodoliths in laboratory microcosms, so were newly discovered congregations of haptophyte cells. Haptophytes are widespread microalgae that are often very abundant in diverse marine habitats with most occurring as solitary motile or non-motile forms, and with a few forming colonies or short filaments^40^,^41^,^42^. Haptophytes are characterized by the presence of a unique organelle called a haptonema, a short flagellum-like appendage, inserted between two smooth flagella^43^. A *Lithothamnion* sp. 2 rhodolith (Fig. 4A) from a Ewing Bank microcosm was used to establish cultures from single cells that were removed from the coralline’s interior (see brown/golden patches of cells, Fig. 4B). A mechanical ultra micropipette under the microscope objectives was used to precisely sample the endolithic stages. Non-flagellated cultures were then successfully established (Fig. 4C,D, Fig. S3) from the single endolithic cells following their isolation. Cultured cells growing on glass slides produced the aggregation of organic material (Fig. 4D). These non-flagellated cells then rapidly developed into free-living, flagellated haptophytes showing two apically inserted flagella and a rudimentary haptonema characteristic of the phylum (Fig. 4E). To further confirm the identity of this haptophyte, total genomic DNA was amplified with REPLI-g from single cells (Fig. 4D,E) grown in cultures that were established from two endolithic inclusions removed from the interior of *Lithothamnion* sp. 2. The DNA originating from the interior and from cells on the surface of *Lithothamnion* sp. 2 was processed separately. Generated nuclear 18S and chloroplast-encoded *tuf*A sequences retrieved from the endolithic cells match a GenBank reference accession of *Ochrosphaera verrucosa* (Coccolithophyceae, Prymnesiophycidae, Coccolithales) [occasionally referred to as *Hymenomonas globosa* (Magne) Gayral & Fresnel], a common coastal haptophyte.

Although the rhodolith surfaces were not treated externally or cleaned, the source of the cellular inclusions is truly endolithic since rhodoliths immediately preserved in silica gel in Ziploc bags upon collection *in situ* contained the endolithic stages that were subsequently confirmed by whole genome amplification using REPLI-g on single cells, and GenBank BLASTing of the DNA sequences. The *Prorocentrum* and *Ochrosphaera* endolithic cells found within the calcium carbonate-lined cell lumens of *Lithothamnion* rhodoliths are previously unreported benthic life history stages in both dinoflagellates and haptophytes, respectively. We hypothesize that the occurrence of endolithic stages inside rhodolith-forming coralline algae is widespread in other bentho-phytoplanktonic taxa as well. We understand that such life history stages may be alternative stages in the life history of *Prorocentrum* that may not always be essential for the completion of its life history and thus may not always be present in the marine environment. Unlike cysts, the endolithic cell aggregations that have been discovered are not surrounded by thick walls. This may be superfluous since the endolithic stages are already enclosed by CaCO_3_ from the coralline. It is interesting to note that *Ochrosphaera* is a member of the Coccolithales, an order of haptophytes characterized by elaborate calcium carbonate scales, called coccoliths in the free-living taxa. Perhaps the production of coccoliths in certain taxa is only temporary^44^.

Based on the present findings, we newly hypothesize that rhodoliths, as calcium carbonate substrata, function as temporary reservoirs for bloom-forming microalgae, including dinoflagellates and haptophytes. The endolithic life history stages in microalgae may be much more widespread than is currently understood and require further attention to assess the universality of endolithic stages within bloom-forming microalgae across multiple algal phyla in marine ecosystems worldwide. For example, *Ochrosphaera verrucosa* has been detected by *tuf*A metabarcoding in reef substrata from geographically isolated reef habitats of Okinawa, Japan and the Florida Keys, USA^27^. In the Pacific and Atlantic oceans, respectively, and thus appears universally present in the CaCO_3_ endolithic niche in the tropics. Endolithic stages may maintain reservoirs of bloom-forming species that allows their seasonal or irregular bloom formation, and survival during or following drastic environmental and ecological shifts, natural or anthropogenic (i.e. the DWH oil spill).

If microalgal associations with rhodoliths are a common feature, such associations could explain some of the enhanced larval settlement of species on rhodolith surfaces in which microalgae on the coralline surfaces could be used as a food resource by early settlers. Most studies on corallines enhancing settlement have been unable to definitely differentiate between whether the coralline cue or a microalgal cue drives settlement, and research on scallops^45^ and on abalone^46^ suggested that benthic diatoms could also be a food source or settlement cue.

The present research has identified a new habitat for enhanced biodiversity (internal to the coralline thallus) which is novel and different from the typical internal hollow locations of rhodoliths^47^. Previous 16S studies of corals have shown that coral symbionts such as *Symbiodinium* dinoflagellates^48^ are present within the coral tissue (mesenteries) for at least part of their life cycle, as do the apicomplexan parasites/Chromerida^49^,^50^, but these cells are not present in the calcium carbonate skeleton.

The point of entry in the thallus and later exit of the newly found life history of dinoflagellate and haptophyte life history stages are currently unresolved and purely speculative. Because corallines have the capacity to slough off their external cell layers (epithallial cell layers), including parts of their conceptacles (reproductive structures), that can become replenished with a unique type of intercalary meristems, it is possible that the microalgal life history stages may passively become surrounded by new surface cell layer growth of the corallines, or perhaps via the large pit connections that approximate the cell width of the endolithic stages. Faust^31^,^32^ noted that upon trying to culture *Prorocentrum lima* from free-living cells found on top of coral rubble and in mangrove sediments, their growth stopped in axenic media. We speculate that the endolithic populations are not permanent residents of rhodoliths but are transient life history stages (potentially resting stages) that may form blooms once released in the water column from the rhodolith’s interior following abrasion or sloughing off of the coralline’s surface cell layers. Alternatively, microborers and macroborers could also be responsible for releasing these cellular inclusions by opening burrows to the water column. This is a fascinating aspect of the newly discovered association of endolithic life history of benthic microalgae with crustose coralline cells that we hope to resolve in future studies or stimulate other research groups interested in the bentho-planktonic coupling of microalgal species.

## Conclusion

Many planktonic organisms are known to possess benthic stages (e.g., cysts) that enable them to persist through adverse conditions^51^,^52^,^53^,^54^. Here we demonstrate that benthic microalgae are endolithically associated within rhodolith cells for parts of their life cycle and hypothesize that this phenomenon is widespread in the marine environment. The study of endolithic microalgal communities may help resolve numerous life histories; for instance, the Haptophyta reported in the present study matched *Ochrosphaera verrucosa* Schussnig AM502964, a common Coccolithophyceae in coastal waters for which all stages in its proposed haplo-diplontic cycle have not yet been observed^43^, and endolithic life history stages were previously unknown for *Prorocentrum lima*. We contend that numerous taxa may lie in the endolithic niche and remain to be documented. The present discovery of unsuspected endolithic stages of microalgae may harbor important implications for understanding and predicting the onset of phytoplankton blooms, including those that form harmful algal blooms (HABs). Our research thus suggests a new ecologically important function for rhodoliths as being remarkable marine benthic microhabitats.

## Methods

### Experimental Design

This includes collection of *Lithothamnion* sp. 1 and sp. 2 rhodoliths and establishment of microcosms with the collected rhodoliths and *in situ* seawater collected from same depth and location. Rhodoliths from the microcosms were used for light microscopy (LM), scanning electron microscopy (SEM), field emission SEM (FESEM), fluorescent microscopy, whole genome amplification using REPLI-g on a single cell, DNA sequencing and DNA analysis.

### Field collections

Deepwater rhodolith collections representing two undescribed species of crustose coralline red algae, *Lithothamnion* sp. 1 and sp. 2 (Hapalidiaceae, Hapalidiales, Rhodophyta), were collected at Ewing Bank (vicinity of 28°05.7′N, 91°01.2′W) offshore Louisiana in the northwestern Gulf of Mexico aboard the *R/V* Pelican, a UNOLS research vessel operated out of LUMCON, Cocodrie LA. Rhodoliths were retrieved using a Hourglass-design box dredge^55^ with minimum tows (usually 10 minutes or less) at depths ranging from 55–70 m. For additional samples, see Table S1. Water samples and environmental factors (e.g., temperature, salinity, photosynthetically available radiation (PAR)) were collected *in situ* using a CTD/Rosette system with sensors and Niskin bottles aboard ship. Samples were initially stored on-site by location in containers filled with seawater collected *in situ* from the same depth and site of the sampled rhodoliths using the onboard CTD water sampling rozette. Samples were kept aerated on board ship for the duration of the trip (2–4 days) and immediately transferred into microcosms, filled with *in situ* collected seawater, located in our laboratory at UL Lafayette 2–5 hours upon return to the laboratory. A subsample of the rhodoliths were also immediately upon retrieval placed in Ziploc bags filled with the desiccant silica gel for long-term preservation for subsequent DNA analysis and microscopy studies.

The two species of *Lithothamnion* investigated in the present study are currently undescribed and both are new to science (Joseph Richards, unpubl. data). In the current literature *Lithothamnion* sp. 1 (LAF6521) that includes the *Prorocentrum* (dinoflagellate) cellular inclusions goes under the name *Lithothamnion occidentale* (Foslie) Foslie which is not correct upon comparative analysis of type material of *L. occidentale* from the US Virgin Islands. *Lithothamnion* sp. 2 that includes the *Ochrophaera* (haptophyte) cellular inclusions also represents an undescribed species.

### Establishment of microcosms

A series of 75-liter closed microcosm tanks, established from a subset of samples from Ewing Bank, were each equipped with a SeaClone 100 protein skimmer (Instant Ocean ^®^, Blacksburg, Virginia), water jets (MJ2000) and 600 lumen lights (FugeRay Unibody) ultra slim aquarium LED light plus Moonlights, Finnex, USA). The protein skimmer provided filtration and a flow of 1,200 liters per hour. The LED photosynthetically available radiation (PAR) in the microcosms^26^ was about 30 micromol (μmol) photons per square meter (m^2^) per second, a measurement approximating *in situ* light PAR or irradiance levels measured with a LI-COR Biosciences (Lincoln, Nebraska) biospherical PAR sensor incorporated in a CTD (for conductivity, temperature, and depth) rosette and water sampler. Each of the closed microcosms was initially filled with *in situ* collected water with CTD rosette Niskin bottles. For some tanks, collected seawater was used untreated, whereas for others, it was sterilized with an ultraviolet filter (Aquanetics Systems, San Diego, California). Systems were maintained at approximately 10 hrs light/14 hrs dark cycle at 24 °C, the same temperature measured in the field at 55 m depth in late summer. Microcosms representing separate sampling sites were filled with a random arrangement of rhodoliths and their respective *in situ* seawater, and/or augmented with sterilized seawater. Deionized water was used to counteract evaporation within the microcosms. Rhodolith vouchers are deposited in the Algal Herbarium of the University of Louisiana at Lafayette (LAF). All algal collections from each trip and from each microcosm were sorted, identified to species level, archived, and deposited at LAF. Subsamples of rhodoliths collected on board ship were also immediately placed in Ziploc bags filled with the desiccant silica gel for long-term preservation for subsequent DNA analysis and microscopy studies. SEM work was also done on these desiccated rhodoliths that included the cellular inclusions. This represents strong evidence that these inclusions are present *in situ* and were not introduced to our microcosm tanks as “contamination”. With regard to any ability of the individuals to be ‘contaminated’ while the rhodoliths were kept in tanks and other macroflora were growing, there were no ‘coastal’ microcosms maintained in the laboratory as a potential source of contamination.

### Light microscopy (LM)

The Ewing bank microcosms were sampled once a month for 12 months. The water samples were examined with an Olympus BX60 compound microscope and photos of free-living dinoflagellates were taken with a Canon PowerShot A3300 camera. Rhodolith surfaces and interiors were cross-sectioned or cut open with straight-edged razor blades or cracked open with a hammer, then viewed under a Zeiss Stemi 2000-C or Olympus SZ61 stereomicroscope. Free-living (swimming) dinoflagellate cells were captured from the microcosm water using a transfer pipette with a thin tip and viewed under the stereomicroscope. Cells captured from within the rhodoliths required a micromanipulator and a compound microscope with a SLMPlan 50X/0.45 objective. Cells retrieved from both the microcosm and the inside of the rhodoliths were cultured, and the taxonomic identity of these stages was confirmed by whole genome amplification using REPLI-g on a single cell.

### Sample preparation for SEM and FESEM

Rhodoliths were prepared for SEM by subdividing whole rhodoliths into fragments that were subsequently preserved in silica gel. Rhodoliths of interest were placed into a folded sheet of paper and underwent short bursts of directed force with a hammer. A variety of microorganisms were found on the surface of various rhodoliths including associated dinoflagellates. Dinoflagellate cells were isolated from rhodolith microcosms and placed on top of coverslips treated with poly-D-lysine^56^ and allowed to settle. Attached cells were then fixed for a minimum of one hour in either Trumps fixative (Electron Microscopy Sciences) or deionized water with 3% sodium chloride, 2.5% glutaraldehyde and 4% paraformaldehyde. The cells were then rinsed and placed through a graded ethanol series to 100% acetone and chemically dried with hexamethyldisiloxane (HMDS). Rhodolith fragments and fixed dinoflagellates were then sputter-coated with 5–10 nm of gold and viewed using either the Hitachi 3000S scanning electron microscope (SEM) or a JEOL field emission scanning electron microscope (FESEM), both housed in the UL Lafayette Microscopy Center.

### Fluorescence microscopy

For fluorescence microscopy, thin sections of the rhodolith material were required. Crushing the rhodolith was to no avail because the orientation of the specimen was often lost in fragments small enough to fit within the working distance of the objectives used. Instead, rhodoliths were fixed in Trumps fixative and sent to Wagner Petrographic (www.wagnerpetrographic.com), a dedicated petrographic laboratory that enabled embedding whole pieces of the rhodolith in clear epoxy. Three samples were semi-thin-sectioned (24 × 46 mm polished to ≈30 microns) and polished. A Nikon E600FN epifluorescence microscope with an Olympus digital camera housed at the UL Lafayette Microscopy Center was used to visualize autofluorescing cells with three different wavelengths, i.e. ultra-violet, blue and green light.

### Sample preparation for TEM

A subsample of rhodoliths used for SEM and FESEM were fixed in Trumps fixative (Electron Microscopy Science) for one hour at ~22 °C. The rhodoliths were rinsed in deionized water, followed by incomplete decalcification in a working solution of 22% formic acid and 10% sodium citrate. The decalcification protocol was altered by allowing the reaction to occur for only 3–5 minutes. The coralline thallus floated to the top of the vial (Fig. S1) and was removed. The working-acid solution was removed without disturbing the precipitate at the bottom of the vial (Fig. S1). The precipitate was washed thoroughly with deionized water, fixed in 2% OsO_4_ for 15 minutes, dehydrated, embedded in Spurr’s resin, cut into 1 nm sections, stained using uranyl acetate and lead citrate^57^ and viewed on a Hitachi 7600 TEM microscope.

### Establishment of cell cultures

Cultures were established from single dinoflagellate cells removed from microcosm water samples and following protocols outlined in ref. 58. Cells were placed in filtered microwaved-sterilized natural seawater for 5–7 days; that water was refreshed with modified K-media every two weeks after day 7. Cultures were kept in 60-well petri dishes at 19 °C with indirect natural sunlight, and periodically checked for growth.

A single *Lithothamnion* sp. 2 rhodolith from the Ewing Bank September 2013 microcosms was used to establish cultures from endolithic single cells that were removed from the inside of that rhodolith and subsequently shown to be *Ochrosphaera verrucosa* haptophytes. Nodules taken from two Ewing Bank rhodoliths collected in 2012 and 2013 and maintained in lab microcosms were shaved transversely with a razor blade into a depression slide filled with NSWK (microwave-sterilized Natural seawater K medium). The shavings were allowed to settle upon which the NSWK was refreshed gently until the water was clear. The shavings were viewed with an Olympus BX60 compound microscope and a SLMPlan 50X/0.45 objective. A Sutter 2000 laser based pipette puller was used to produce ultra-micropipettes. The pipettes were controlled using a micro-manipulator and suction was controlled using a one-way valve and a transfer pipette. The total apparatus included the microscope with Plan objective and an ultra-micropipette with micromanipulator that allowed us to remove single cells from within the rhodolith shavings. These cells were cultured in NSWK. Cells originating from the inside of the rhodoliths from the 2013 Ewing Bank microcosms divided to produce three separate cultures. The cells originating from the 2012 culture did not divide.

### Molecular data acquisition

#### Whole sample DNA extraction

The subsurface of four rhodoliths was sampled (samples taken from microcosms established from *Lithothamnion* sp. rhodoliths collected from Ewing Bank in October 2012) using a sterile 1.6 mm (1/16″) bit mounted on a Flex-Shaft Attachment (Model 225) powered by a Dremel 3000 rotary tool (Dremel^®^, Racine, WI, USA), carefully avoiding the surface of the rhodolith. DNA was extracted from this sample using the DNeasy Plant Mini Kit (Qiagen, Valencia, CA, USA) following the manufacturer’s instructions.

#### Single cell DNA extraction

A single cell DNA extraction technique (single cell PCR) was modified from Ki *et al*.^58^. Dinoflagellates were first isolated from established cultures using a small subsample of a culture placed on a slide and viewed under the Zeiss Stemi 2000-C dissecting scope. Individual cells were isolated by micropipetting a small volume (1–3 μm) of culture media containing an individual cell. To ensure it was the only cell recovered, the isolated cell was then moved through three pools of 40 μl of TE buffer on a slide, and verified on the Olympus BX60 microscope. The dinoflagellate cell was then retrieved in 2 μl of the surrounding TE buffer and placed in 200 μl PCR tube with 5 μl Proteinase K (200 mg/ml) and covered with a layer of sterile mineral oil. The sample was then loaded into the BioRad T100 thermocycler for a 50 minute cycle at 55 °C degrees; the subsequent amplification by PCR occurred within the same tube. When single cell PCR was unsuccessful we also used a Qiagen REPLI-g single cell kit to extract DNA from single cells removed from both the inside of the *Lithothamnion* rhodolith and from its surface. The REPLI-g protocol was modified by adding a 5-minute incubation period at 95 °C before the denaturing step (65 °C for 10 minutes). All other steps occurred according to the manufacturer’s directions. This provided genomic DNA from the individual cells. PCR with specific primers then followed.

### DNA amplification and sequencing

C*ob1*, partial 18S and *tuf*A barcode were amplified by PCR using MangoTaq^tm^ DNA Polymerase (Bioline, Taunton, MA, USA) with the cycle and dinoflagellate-specific *cob1* primers described in Lin *et al*.^59^, and haptophyte-specific 18S primers^43^ and degenerate *tufA* primers^27^. The resulting PCR products were then gel-purified followed by gene cycle sequencing with the BigDye^®^. Terminator v3.1 Cycle Sequencing Kit (Life Technologies, Grand Island, NY, USA). Cycle sequencing reactions were then purified using Ethanol/EDTA precipitation. The resulting dried precipitated DNA was then resuspended in HiDi^tm^ formamide (Life Technologies, Grand Island, NY, USA), heat-denatured and sequenced on the departmental ABI 3130xl Genetic Analyzer at UL Lafayette.

### DNA assembly and analysis

The resulting sequences were then assembled on Sequencher v 5.1 (GeneCodes^®^ Ann Arbor, MI, USA). The assembled sequences were run through BLASTn (http://blast.ncbi.nlm.nih.gov/Blast.cgi) and the nearest hits were downloaded from the public NCBI database and used to establish the data sets. The resulting dinoflagellate data set for *cob1* consisted of 34 Prorocentrales and 1 outgroup, *Gymnodinium simplex* (Suessiales)^60^. The resulting haptophyte dataset for 18S consisted of 20 Coccolithales and 1 outgroup, *Dicrateria inornata* (Prymnesiales). The resulting haptophyte dataset for *tuf*A consisted of 19 Coccolithales, 1 Prymnesiales (*Imantonia rotunda*) and 3 outgroups belonging to *Pavlova* (Pavlovales). The following GenBank numbers represent:

*Prorocentrum lima (cob1*): endolithic stage: KX643362; free-living stage: KX643361, KX643363, VKX643364, KX643365, KX643366

*Ochrosphaera verrucosa* (18S): endolithic stage: Ewing Bank: KX664103, KX664104; free-living stage: KX664105; (*tuf*A): http://dx.doi.org/10.5061/dryad.6cj8h.

The sequences of each dataset were then aligned in Mega v 5.2.2^61^. The final alignments were further analyzed using Partitionfinder^62^ to determine the best fitting model of evolution and optimum data partition. The analyses resulted in the selection of the General Time Reversible model plus gamma with a proportion of invariable sites applied separately to each codon position for the protein-encoding genes *cob1* and *tuf*A on the basis of the three information criteria, i.e. Akaike information criterion corrected (AICc), Akaike information criterion (AIC) and Bayesian information criterion (BIC). The alignments of the *cob*1 and *tuf*A datasets were analyzed separately by Maximum likelihood (ML) as implemented by RAXML v 2.4.4^63^ with the above models and partition scheme with 1000 restarts to find the tree with the lowest likelihood score and 1000 Bootstrap (BS) replications. The 18S dataset was analyzed by Maximum likelihood (ML) as implemented by RAXML v 2.4.4 without partitioning.

## Additional Information

**How to cite this article:** Krayesky-Self, S. *et al*. Eukaryotic Life Inhabits Rhodolith-forming Coralline Algae (Hapalidiales, Rhodophyta), Remarkable Marine Benthic Microhabitats. *Sci. Rep.* **7**, 45850; doi: 10.1038/srep45850 (2017).

**Publisher's note:** Springer Nature remains neutral with regard to jurisdictional claims in published maps and institutional affiliations.

## Supplementary Material

Supplementary Information
