# Supplementary material for: Eukaryotic Life Inhabits Rhodolith-forming Coralline Algae (Hapalidiales, Rhodophyta), Remarkable Marine Benthic Microhabitats
Source: Sci Rep. 2017 Apr 3;7:45850. doi: 10.1038/srep45850 (PMC5377461; doi:10.1038/srep45850)
Supplement: Supplementary Information [file srep45850-s1.pdf]

Supplementary Materials for

Eukaryotic Life Inhabits Rhodolith-forming Coralline Algae (Hapalidiales, Rhodophyta), Remarkable Marine Benthic Microhabitats

Sherry Krayesky-Self*,William E. Schmidt, Delena Phung, Caroline Henry, Thomas Sauvage, Olga Camacho, Bruce Felgenhauer, Suzanne Fredericq

Corresponding author. Email: slk5014@louisiana.edu

This file includes:

Table S1

Figures S1, S2, S3 and legends

Table S1; Collection, locality data. Abbreviations: NWGMx (NW Gulf of Mexico), NEGMx (NE Gulf of Mexico)

Litho-thamnion Sample	LAF Identifica-tion Number	Preservation mode	Location	Lati-tude	Longi-tude	Depth (meters)	Colle ction date
1	4096-A	Silica gel	NWGMx, Ewing Bank	28°05. 935	91°02.129	56.5-56	29-viii-2011
2	4096-B	Silica gel & decalcified for SEM & TEM	NWGMx, Ewing Bank	28°05.935	91°02.129	56.5-56	29-viii-2011
3a	6573	Microcosm IV established in 2012	NWGMx, Ewing Bank microcosm IV	27°57.08	92 ^{o}01.03	54-55	26-viii-2012
3b	6573	Microcosm IV established in 2012	NWGMx, Ewing Bank microcosm IV	27°57.08	92 ^{o}01.03	54-55	26-viii-2012
4	10-19-13-3	Microcosm III established in 2013	NWGMx, Ewing Bank microcosm III	28°04.634	91°02.014	85-90	19-x-2013
5	8-24-12-4-1	Silica gel & decalcified for SEM & TEM	NWGMx, Sackett Bank	28°38.201	89°32.916	64-80	24-viii-2012
6	8-25-12-4-3	Silica gel	NWGMx, Sackett Bank	28°38.202	89°32.917	64-80	25-viii-2012
7	10-17-13-4	Silica gel	NWGMx, Sackett Bank	28°37.984	89°33.005	64.80	17-x-2013

8	9-7-14-7	Microcosm I established in 2014	SEGMx, vicinity of Dry Tortugas, FL microcosm VII	28°38 .202	28°38 .202	42-43	7-ix-2014
9	1437	Silica gel	NEGMx, Florida Middle Grounds	28°10 .27	84°02 .07	42-43.2	5-vii-2006
10	01-14-11-1-16	Silica gel	Isla Canal de Afuera, Pacific Panama	7 °42.877	81°36. 55	~15	14-i-2011
11	12-4-10-5-2	Silica gel	NWGMx, Ewing Bank	28°5. 898N	91°1. 860W	53-56	4-xii-2010
12	8-29-11-1-6	Silica gel	NWGMx, Ewing Bank	28°05 .757	91°00 794	61-68	29-viii-2011
13	4100 A	Silica gel	NWGMx, Ewing Bank	28°05 .826	91°01 .555	56.5-56.7	29-viii-2011
14	4100 B	Silica gel	NWGMx, Ewing Bank	28°05 .826	91°01 .555	56.5-56.7	29-viii-2011
15	4100 C	Silica gel	NWGMx, Ewing Bank	28°05 .826	91°01 .555	56.5-56.7	29-viii-2011
16	4091 A	Silica gel	NWGMx, Ewing Bank	28°5. 935	91°02 .129	56.5-56	29-viii-2011
17	4091 B	Silica gel	NWGMx, Ewing Bank	28°5. 935	91°02 .129	56.5-56	29-viii-2011
18	4091 C	Silica gel	NWGMx, Ewing Bank	28°5. 935	91°02 .129	56.5-56	29-viii-2011
19	11-16-12-5-1	Silica gel	NWGMx, Ewing Bank	28°05 .871	91°02 .054	54-79	19-xi-2012
20	1572	Silica gel	NWGMx	27°98 .947	91°39 .382	66-75	21-viii-2008
21	1663	Silica gel	NWGMx	28°38 .090	89°33 .510	66-75	21-viii-2008
22	6558	Silica gel	NWGMx	59 97 27	39 41 91	63-65	21-viii 2008
23	1662	Silica gel	NWGMx	27'58. 165	91'39 .522	65-69	21-viii-2008
24	5-10-12-1-12	Silica gel & decalcified for	Caribbean Panama	82°25 12	9°347 3	~15	10-v-2012

		SEM & TEM					
25	6521	Silica gel	NWGMx, Ewing Bank	28°05 .845	91°01 .817	54-58	16-xi-2012
26	8-29-11-3-2	Silica gel, & sections by Wagner Petrographic	NWGMx, Ewing Bank	28°05 .689	91°01 .915	55	29-viii-2011

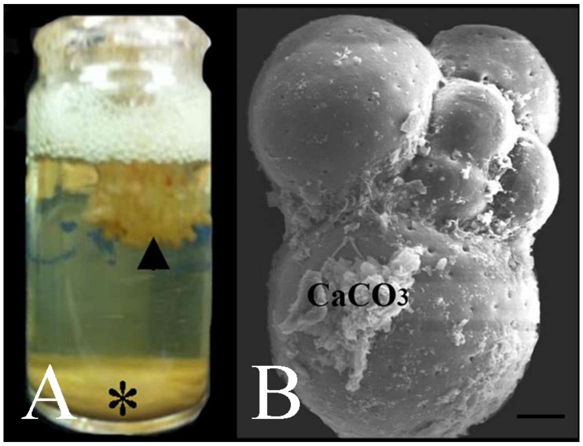

Figure S1: **A)** Vial containing remnants of a rhodolith [sample 5] following incomplete decalcification; coralline algal surface cell layers (arrow) and precipitate (*) from coralline medullary cells. **B)** Clusters of cells with thecal pores isolated from *Lithothamnion* sp. debris following decalcification. Sample from Ewing Bank microcosm established on August 2012, scale bar = 10 µm.

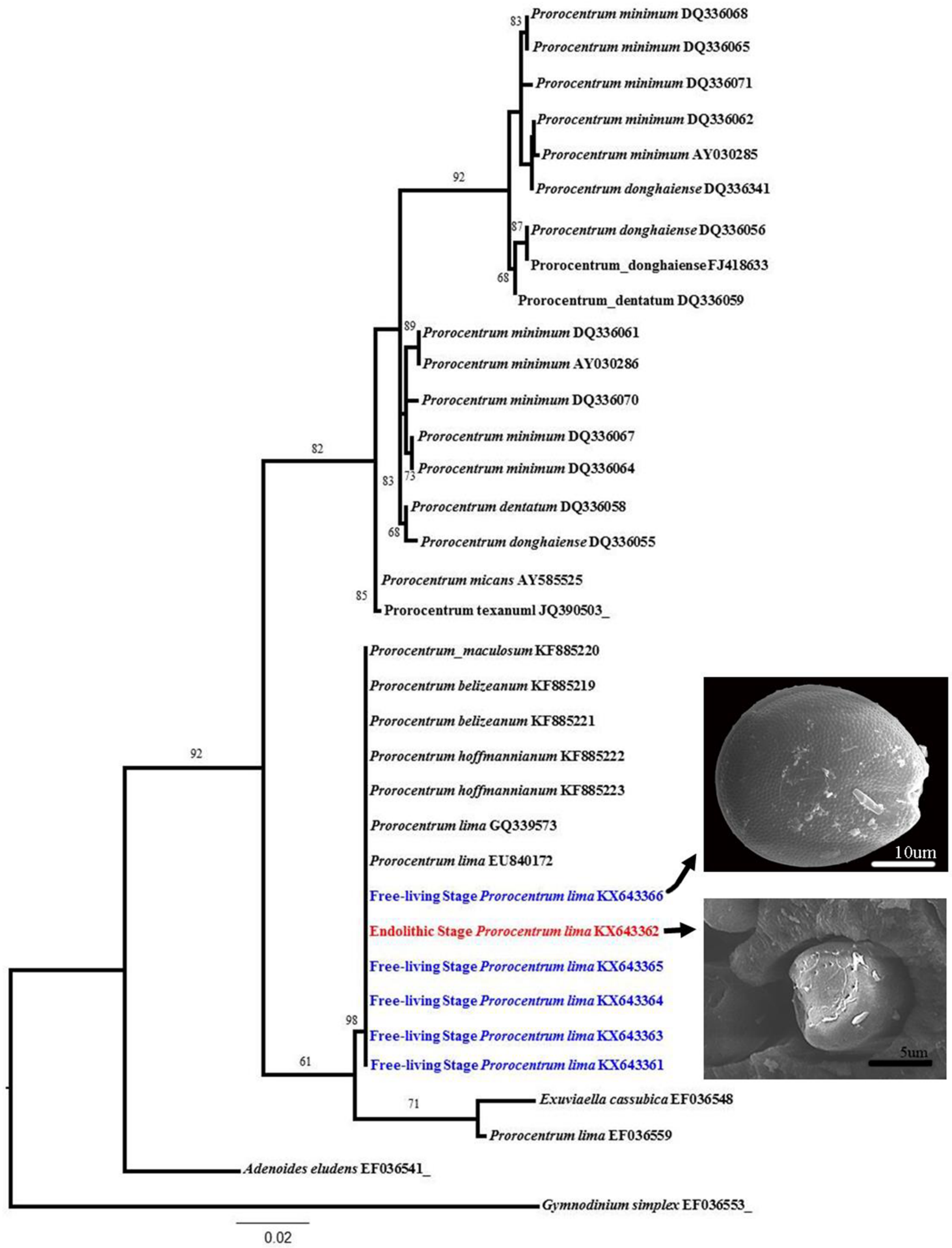

Figure S2: *Prorocentrum* phylogeny produced with RAxML for the mitochondrial-encoded gene *Cob1*. The tree highlights and confirms the molecular identity of endolithic cellular inclusions (label highlighted in purple) within a rhodolith-forming *Lithothamnion* sp. 1 and free-living stages (label highlighted in blue) as *P. lima*. All samples were obtained from a Ewing Bank microcosm. Additional *Prorocentrum* and outgroup sequences were downloaded from GenBank for context. Scale bars: 10 µm (for free-living cell) & 5 µm (for endolithic cell).

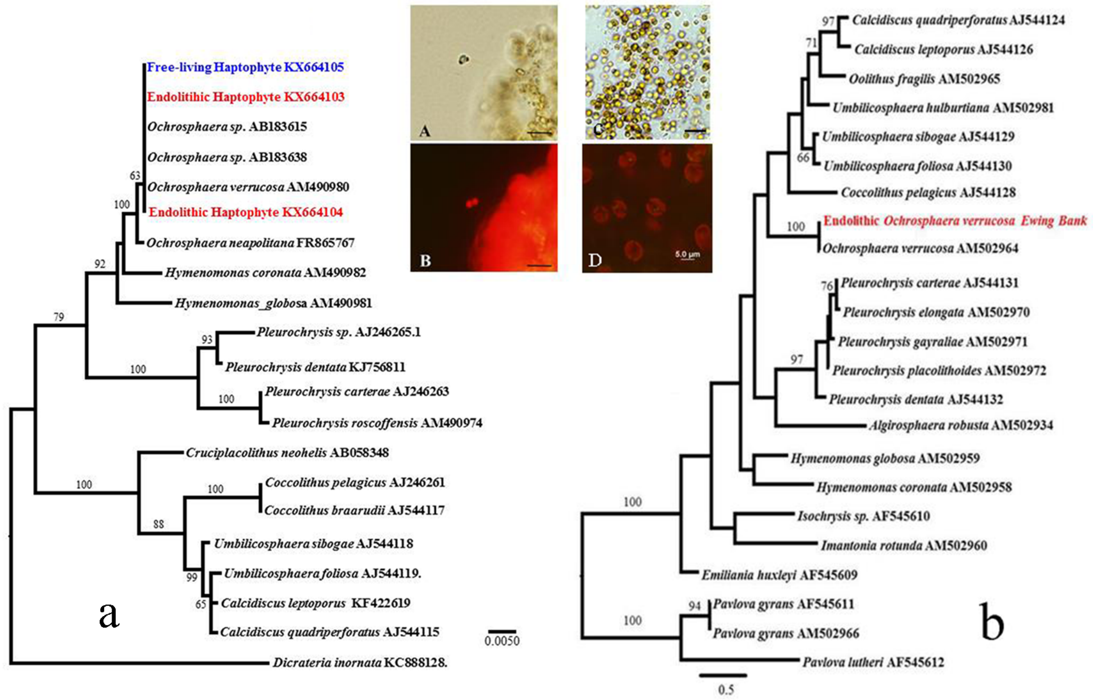

Figure S3: Haptophyta phylogeny produced with RAxML for the nuclear small subunit 18S (a) and chloroplast-encoded gene *tuf*A (b). The trees highlight and confirm the molecular identity of endolithic cellular inclusions (label highlighted in blue) sampled with a mechanical ultra micropipette from a rhodolith-forming *Lithothamnion* sp. 2 (see Material and Methods in Supplementary Materials) and free-living stages (label highlighted in blue) as *Ochrosphaera verrucosa.* Endolithic samples collected from a NW Gulf of Mexico (Ewing Bank), microcosm and cultured into free-living stages (**Figs A-B)**; additional free-living stages were collected from a SE Gulf of Mexico (vicinity of Dry Tortugas, FL) microcosm (**Figs C-D**). Figs B and D are fluorescent micrographs of the samples illustrated in A and C with light microscopy. Additional Haptophya sequences (ingroup and outgroup) were downloaded from GenBank for context. Scale bars: A = 8 µm, B = 8 µm, C = 10 µm, D = 5 µm.
